# Supplementary material for: Does credit rating agency reputation matter in China’s local government bond market?
Source: PLoS One. 2022 Sep 20;17(9):e0274828. doi: 10.1371/journal.pone.0274828 (PMC9488777; doi:10.1371/journal.pone.0274828)
Supplement: Attachment — Submitted filename: Response to Reviewers.pdf [file pone.0274828.s003.pdf]

Does credit rating agency reputation matter in China's local government bond market?

Changqian Xie, Rubi Ahmad, Eric H. Y. Koh*

The entire manuscript has been native-speaker proofread by a professional company to make sure our expression more accurate and clear. We also checked our reference list, and there is no retracted article. However, six articles were published in the Chinese language, and we have marked in the reference list. The point-by-point responses to the reviewers' comments are as follow. If there is anything needing further improvement, please notify use without hesitation. We will complete the modification as soon as possible.

Reviewer #1: The paper has improved substantially. I advise the authors to employ a good professional copy-editor to improve the exposition of the paper.

Response:

Thank you very much for your kind suggestions. We sent our manuscript to a professional institution and completed the proofreading. We hope this revised version can meet the requirement of language.

Reviewer #2: The authors have responded adequately almost all my comments. However, I have a concern regarding their response to my comment 3. As the authors are surely aware, there is at least one recent scandal that led to a punishment of the Dagon agency in 2018 (see https://www.bnnbloomberg.ca/china-rating-firm-banned-as-regulators-cite-fake-info-chaos-1.1125717#:~:text=China%20Rating%20Firm%20Banned%20as%20Regulators%20Cite%20Fake,punishment%20ever%20doled%20out%20a%20ratings%20company). This implies a lower reputation of this agency compared to the others. There are also cases of international credit rating misbehaviour outside China, but well known to international markets. All these cases (at least one of them) can be used to test the effect of reputational shocks.

Response:

We apologize for that our unclear explanation made you misunderstand. First, we agree that the punishment of the Dagong agency in 2018 implies

a lower reputation for this agency. However, the Dagong agency in our study has been classified in the low reputation group. It is worth noting that we only divided all agencies into two groups (i.e., high and low reputation groups), instead of into more groups with more details, such as the groups with low and lower reputations. Therefore, the influence of this punishment on the reputation of Dagong agency is not suitable to be considered in this study.

Second, when it comes to other cases of international credit rating misbehavior, all the cases belong to the events that happened in different markets and in different countries. If these cases are included in our manuscript to study their influence, then the analysis will undoubtedly result in some complicated spillover effects. Therefore, previous literature (Livingston et al.,2018; Hu et al.,2020) has not completed a similar analysis, either.

Lastly, even if there is any event that can influence the reputation of the agencies in China's local government bond market, it cannot become the criterion to evaluate whether or not these agencies are in the correct groups with high and low-reputation. As you have mentioned, the case of Dagong agency can only make its reputation lower. However, it is not related to its original reputation being high or low. We believe that the results of our baseline regression and robust checks can demonstrate the correction of dividing the current high- and low-reputation groups. In the baseline regression, the results listed in Table 8 show that the coefficients of *Reputation_CRA* to *Risk Premium 5* and *Risk Premium 0* are negative and statistically significant. According to the reputation certification hypothesis, only those with high reputation can help reduce bond risk premium. Therefore, the results verify that our selection of high-reputation group enjoys high reputation. If our selection is wrong or opposite (i.e., current high reputation is essentially low reputation, and current low reputation is essentially high reputation), then the coefficients should be positive. Similarly, the result of the second-stage Heckman model (Table 9) shows that the coefficient of *Reputation_CRA* to *Risk Premium 5* is –10.0667. Coefficients of *Reputation_CRA* to *Risk Premium 5* to *Risk Premium 0* in the DID regression results in Table 11 are –15.2346 and –14.7596, respectively. In machine learning, differences between the factual set (high reputation) and counterfactual set (low reputation) are –0.103bp and –0.0561bp, respectively. When excluding negative risk premium observations, Table 14 shows that coefficients of *Reputation_CRA* to *Risk Premium 5* to *Risk Premium 0* are –5.8774 and –6.4877, respectively. When using the subsample for robustness check, the two coefficients are –9.3151 and –11.7342, as shown in Table 15. All preceding coefficients are negative, demonstrating the correction of our selection. Hence, we think it's not

necessary to include additional analysis on reputation shock.

Thank you very much for your kind comments and hope our explanations can release your concerns.
