# Supplementary material for: Does credit rating agency reputation matter in China’s local government bond market?
Source: PLoS One. 2022 Sep 20;17(9):e0274828. doi: 10.1371/journal.pone.0274828 (PMC9488777; doi:10.1371/journal.pone.0274828)
Supplement: Attachment — Submitted filename: Response to Reviewers.pdf [file pone.0274828.s004.pdf]

Does credit rating agency reputation matter in China's local government bond market?

Changqian Xie, Rubi Ahmad, Eric H. Y. Koh*

This revised manuscript mainly has the following modifications: (1) the second hypothesis development is revised; (2) we added more content to discuss the economic significance of our results throughout the paper. All the main modifications have been highlighted in the document. We also sent the modified content to native-speaker proofreading. The point-by-point responses to the reviewer's comments are as follow. If anything needs further improvement, please notify us without hesitation. We will complete the modification as soon as possible.

Reviewer #1: There are two remaining issues for the authors to address: first, the hypothesis H2 needs to be developed in more depth and breadth. Second, the economical significance of the results need to be discussed throughout the paper.

Response:

Thank you very much for your kind suggestions. First, we apologize that the explanation and development of hypothesis 2 in the previous version were too brief. In this revised version, we reviewed more previous research and revised the development of hypothesis 2 to meet your requirement (presented on Page 7-Page 8). Second, we added more contents to discuss the economic significance of our results throughout the paper, which are highlighted in the manuscript as follows: 1) Abstract (presented on Page 1); 2) Introduction (presented on Page 2); 3) Baseline regression analysis (presented on Page 15); 4) Endogeneity concern: difference-in-differences regression (presented on Page 21); 5) Conclusion (presented on Page 30). In addition, we sent our manuscript to a professional institution to proofread the revised content again.

Thank you very much for your kind comments again and hope our modifications can meet your requirements.

Reviewer #2: I find the authors' response to my comment on reputational shocks satisfactory. However, I encourage the authors to explore this

avenue in their future research, as I believe that such an analysis can yield interesting and useful results.

Response:

Thank you very much for all your previously valuable suggestions and recognition of our revised work. We would like to set the mentioned analysis as our future research focus according to your suggestion.

Thank you very much again!
